# Osteokines and Bone Markers at Rest and following Plyometric Exercise in Pre- and Postmenopausal Women

**DOI:** 10.1155/2020/7917309

**Published:** 2020-10-21

**Authors:** Katlynne Nelson, Rozalia Kouvelioti, Alexandros Theocharidis, Bareket Falk, Peter Tiidus, Panagiota Klentrou

**Affiliations:** ^1^Department of Kinesiology, Brock University, Ontario, Canada; ^2^Centre for Bone and Muscle Health, Department of Kinesiology, Faculty of Applied Health Sciences, Brock University, 1812 Sir Isaac Brock Way, St. Catharines, Ontario, Canada

## Abstract

The effect of plyometric exercise on bone biomarkers has been studied in pediatric and young adult populations in order to better understand how exercise influences bone homeostasis. However, there are no such data in postmenopausal women, a group characterized by an uncoupling of the bone resorption-formation cycle. This study examined the serum concentrations of sclerostin, dickkopf-1 (DKK1), c-terminal crosslinking telopeptides of type I collagen (CTXI), and procollagen type I amino-terminal propeptide (PINP) at rest and following a single bout of plyometric exercise in 20 premenopausal (23.1 ± 2.3 years) and 20 postmenopausal women (57.9 ± 4.3 years). The exercise consisted of 128 jumps, organized into 5 circuit stations. Blood samples were obtained prior to and 5 min, 1 h, and 24 h postexercise. At rest, postmenopausal women had significantly higher sclerostin and CTXI, but lower DKK1 than premenopausal women. Sclerostin increased 5 min postexercise only in the premenopausal group. DKK1 decreased 24 h postexercise in the premenopausal women while it decreased 1 h postexercise in the postmenopausal women. In both groups, CTXI did not change across time and PINP decreased 5 min and 1 h postexercise (*p* < 0.05). The PINP/CTXI ratio decreased 5 min and 1 h postexercise then significantly increased 24 h postexercise only in premenopausal women. These results indicate that although plyometric exercise is effective in eliciting osteoanabolic effects in younger women; such an effect is not evident in postmenopausal women.

## 1. Introduction

Following menopause, women experience an accelerated decline in bone mass caused by the uncoupling of the bone resorption-formation cycle, when the rate of bone formation by the osteoblasts does not match the rate of resorption by the osteoclasts [1, 2]. This uncoupling is due to the decline in circulating estrogen, which influences the balance of bone remodeling [3, 4]. The changes in bone remodeling that occur with menopause are reflected in increased circulating bone resorption markers like c-terminal crosslinking telopeptides of type I collagen (CTXI) and procollagen type I amino-terminal propeptide (PINP) [5, 6]. These two markers are recommended by the International Osteoporosis Foundation as reference markers of bone turnover for the estimation of future fracture risk and evaluation of treatment effectiveness in clinical settings [7]. PINP is a sensitive marker of dynamic changes in bone formation that reflects changes in new collagen synthesis, while CTXI is a sensitive marker of bone resorption reflecting the rate of collagen breakdown [8]. In addition, the PINP/CTXI ratio has been positively correlated with bone mineral density (BMD) of the hip and spine in postmenopausal women [9].

In contrast to menopause, regular exercise and physical activity are considered beneficial for improving or preserving bone formation and bone mineral density in women, both pre- and postmenopause [10, 11]. High-impact exercise, such as plyometrics, has been shown to be most beneficial for the bone, specifically in young girls and women, due to the high mechanical strain from the high ground reaction forces [10, 12]. However, its role in bone homeostasis postmenopause is still undefined as it is not recommended for individuals at risk or with osteoporosis because of a perceived high risk of fracture. In addition, previous studies examining the acute response of CTXI, PINP, and other bone turnover markers to exercise have produced inconclusive results, with some reporting no effect of exercise and some reporting a transient increase, while others reporting inconsistent changes [10, 13–17]. These studies involve various modes of exercise, different markers, males and females, athletes and nonathletes, and various age groups. None of these studies examined or compared the response of PINP and CTXI to plyometric exercise in younger and older women.

The bone resorption-formation cycle is also regulated by osteokines. Sclerostin and dickkopf-1 (DKK1) are catabolic osteokines that downregulate bone formation through inhibition of the Wnt pathway. Specifically, sclerostin is secreted by osteocytes to inhibit the Wnt pathway, reducing osteoblast activity and, therefore, bone formation [18]. DKK-1 is another Wnt-inhibiting protein, which is expressed by both the osteocytes and the osteoblasts [19, 20]. Elevated serum DKK1 concentrations have been observed to enhance osteoclastogenesis, increasing the activity of the osteoclastic cells [21]. Although it is well established that sclerostin resting levels are significantly higher in postmenopausal women in comparison to their younger counterparts [22, 23], studies comparing resting levels of DKK1 between pre- and postmenopausal women are lacking. Moreover, despite two studies showing higher daily physical activity levels be associated with lower serum sclerostin levels in postmenopausal women, arguably due to the increased mechanical loading on the bones [24, 25], sclerostin has consistently been shown to increase immediately following both high-impact and low-impact exercises in young adults [26–29]. An examination of the acute response of these osteokines to plyometric exercise and in relation to changes in bone turnover markers in older, postmenopausal women as compared to younger, premenopausal women is yet to be performed. Insight into this response has important implications to our understanding of how the bone's response to mechanical loading can contribute to preventive strategies against age-related bone loss.

The purpose of this study was to examine the serum concentrations of osteokines related to Wnt signaling pathway, namely, sclerostin and DKK1, and markers of bone formation (PINP) and bone resorption (CTXI) at rest and in response to a single bout of high-impact plyometric exercise in young, premenopausal and older, postmenopausal women. Based on previous findings, it was hypothesized that at rest, sclerostin, DKK1, and CTXI would be lower while the PINP and PINP/CTXI ratio would be higher in the younger women and that sclerostin would increase immediately postexercise while DKK1 would decrease 24 h postexercise in both groups.

## 2. Methods

### 2.1. Participants

Forty healthy women volunteered to participate in this study: 20 young (18-28 years, premenopausal) women and 20 older (52-68 years, postmenopausal) women. Participants were recruited from the University and broader community based on the following inclusion criteria: body mass index < 30; not taking pharmaceutical agents that directly affect the bone; and had no facture within the last six months. In addition, postmenopausal women had to be at least 2 years postmenopause (self-reported) and should have not been diagnosed with osteopenia or osteoporosis or other conditions for which plyometric exercise was contraindicated (written confirmation from their physician was required). All participants in the premenopausal group self-reported being eumenorrheic and were tested during their follicular phase. All participants agreed to participate in this study by signing a consent form. The study was conducted in accordance with the Declaration of Helsinki and received ethics approval from our institutional Research Ethics Board (REB #14-267).

### 2.2. Study Design and Procedures

The two-day study protocol involved two visits to the laboratory within 24 hours. Participants were asked to refrain from caffeine and alcohol consumption for 8 hours prior to visiting the laboratory and to not perform any vigorous activity for 24 hours prior to their first visit and up to the time of their second visit. All visits to the laboratory were scheduled in the morning between 0800 and 0900 hours, with the specific time for each participant being the same at baseline and at 24 hours, to account for circadian rhythm variations and in fasted state to minimize the influence of food intake on the biochemical markers.

Upon arrival to the laboratory, the participants were informed of the research protocol and asked to sign an informed consent, then completed a medical history questionnaire. Subsequently, a baseline, resting blood draw was performed using venipuncture of the median cubital vein. After the first blood sample, participants were provided with a standardized breakfast consisted of a granola bar, a juice box, and a banana. The breakfast was followed by the following anthropometric measurements: height (cm), weight (kg), waist and hip circumference (cm), jump height (cm), and body composition. The women then performed a plyometric exercise trial followed by 3 postexercise blood samples: 5 min, 1 h, and 24 h postexercise. Participants also completed the Godin-Sheppard Leisure Time Exercise Questionnaire and a Food Frequency Questionnaire.

To examine potential intraindividual differences in the serum levels of the selected biomarkers during the study hours, a subgroup of 5 of the participating women (3 premenopausal, mean age 26.7 ± 0.8 years; 2 postmenopausal mean age 60.5 ± 7.8 years) were invited back to the laboratory for a nonexercise, control trial, which was not part of the original experimental design. During this control trial, 3 resting blood samples were collected from each participant every 30 min, at the same time of day as per the exercise trials.

### 2.3. Exercise Trial

The exercise plyometric session was designed to elicit an impact on the bone through various jumping exercises performed in a circuit, as previously used in our laboratory [14, 28, 30]. It began with 5 min warm-up on the cycle ergometer and dynamic stretching. The participants were familiarized with the exercises through a detailed explanation and a demonstration of each exercise.

The exercise protocol consisted of 128 jumps organized into five circuit exercise stations, with three minutes of rest between stations. The circuit included box jumps, lunge jumps, tuck jumps, single-leg hops, and jumping jacks. Each participant was instructed to rotate through each of the five stations 3 times, i.e., 3 sets of 8 jump repetitions, except for the single-leg hops, which were performed as 2 sets of 8 repetitions on each leg. The box jump height was adjusted to each participant's maximal jump height, averaging 35.0 ± 0.9 cm for the younger women and 26.7 ± 0.7 cm for the older women. The maximal jump height was determined by the average of 3 vertical jumps measured using a vertical jump measuring device. This was to ensure that the ground reaction forces produced from the box jump were relatively similar among participants, without compromising the safety of older participants. The participants were also encouraged to go at their own pace to ensure comfort and reduce the risk of injury during the protocol. The exercise protocol ended with a five-minute cool-down using static stretching.

### 2.4. Measurements

Measurements of body mass and percent body fat (%BF) were taken using the InBody520 bioelectrical impedance analysis (BIA) system (Biospace.228). Participants were asked to be well hydrated before the BIA measures and were asked to void before the measurement. Height was measured using a stadiometer to the nearest 0.1 cm. Jump height was measured using a Vertec Jump Height measurement device to the nearest 1.0 cm. These measures were taken with no shoes and in exercise clothing. To maintain measurement consistency, the same investigator performed all anthropometric measures.

Participants completed the Godin-Shepard Leisure Time Exercise Questionnaire (Godin and Shephard, 1985), and their physical activity levels were assessed by calculating their leisure score index. Participants' habitual nutrient intake was also determined using a food frequency questionnaire (Block 2014.1_6Mo, Nutrition Quest, USA).

### 2.5. Blood Analysis

Four venous blood samples were taken: at rest (preexercise) and at 5 min, 1 h, and 24 h following the exercise from the median cubital vein in the antecubital fossa of each participant using a standard venipuncture technique. At the first time point (i.e., resting sample), 18 mL of blood was collected, and 10 mL of blood was collected at all postexercise blood draws. The serum for the biochemical markers was collected in vacutainers with an SST serum separator. All the SST serum separator vacutainers were allowed to stay in the laboratory for 30 minutes at room temperature before centrifuging. The vacutainers were centrifuged at 3000 × gravity for 15 minutes in a benchtop centrifuge (Allegra ZIR centrifuge, Beckman Coulter, USA) to separate the serum from the red blood cells. The serum was then aliquoted into microcentrifuge tubes and stored at -80°C until analysis.

Serum sclerostin, CTXI, and PINP were measured in duplicate using commercially available immunoassay (ELISA) kits (sclerostin, cat. # DSST00, R&D Systems Inc., CTXI, cat. # E-EL-H0835, Elabscience; PINP, cat. # E-EL-H0185, Elabscience). DKK1 was also measured in duplicate analyzed using Magnetic Luminex Assays (cat. # LXSAHM, R&D Systems Inc.). Our in-house inter- and intra-assay coefficients of variation (CV) for sclerostin, CTXI, PINP, and DKK1 were 7.1% and 7.7%, 6.5% and 6.4%, 2.0% and 8.2%, and 6.3% and 8.2%, respectively. Baseline (i.e., resting) serum estradiol was measured in duplicate using an ELISA kit (cat. # KGE014, R&D Systems Inc.) with an intra-assay CV of 11.7%.

### 2.6. Statistical Analysis

From the 160 blood samples (40 participants × 4 time points), there were missing values due to either a missed blood draw or insufficient amount of blood to run all assays. Participants with more than two missing values were excluded from further analysis; 3 participants were excluded for DKK1, 2 participants for CTXI, and 6 participants for PINP (i.e., 8 participants were subsequently excluded from the PINP/CTXI ratio). No participant was excluded from sclerostin. If a participant had no more than two missing values, each of those missing values was replaced with the group mean value at the corresponding time point (9 of 160 [5%] for sclerostin, 10 of 157 [6%] for Dkk1, 13 of 158 [8%] for CTXI, and 12 [8%] of 154 for PINP). The replacement of missing values with the group mean value is often used in a repeated measure design, as it does not affect the group mean of a particular time point while preserving the rest of these participants' data in the analysis [31, 32]. The data were then assessed for normality using the Shapiro Wilk test, *z*-scores for skewness and kurtosis of ±3, and visual screening of histograms for symmetry. In case of violation of normality, which was the case for sclerostin, CTXI, PINP, and their ratio, the data were log-transformed data for subsequent analysis.

Group differences in mean anthropometric measures, dietary intake, physical activity, and resting biochemical concentrations were tested using independent *t*-tests. A mixed analysis of variance with time as the within-subject variable and group as the between-subject variable was used to examine differences in the response of bone turnover markers and osteokines to plyometric exercise. Significant interactions and main effects for time were further examined using Least Significant Difference post hoc pairwise comparisons. To examine changes over time during the nonexercise control session, we used the Friedman nonparametric analysis of repeated measures due to the small number of participants completing this session. Pearson's correlation analysis was subsequently used to determine associations between variables at rest and between the absolute changes from pre- to postexercise. Correlation analysis was performed on the two groups combined. The statistical significance was set at an alpha level of 0.05, and all statistical analyses were performed using IBM SPSS Statistics 25 (SPSS Inc., Chicago, IL, USA).

## 3. Results

The physical and nutritional characteristics of the participants are presented in Table 1. Postmenopausal women had significantly higher body fat than premenopausal women, but there were no significant differences in physical activity levels and dietary intake (energy, protein, and calcium) between the two groups. In addition, during the nonexercise control trial, the serum levels of all bone markers were stable over time with no significant changes found over time using the Friedman analysis of repeated measures and with the intraindividual CV averaging 9.4% for sclerostin, 5.3% for DKK1, 9.5% for CTXI, and 10.1% for PINP (Table 2). Postmenopausal women had significantly higher sclerostin and CTXI at rest and significantly lower DKK1 and estradiol compared with the premenopausal women (Table 3).

**Figure 1:**
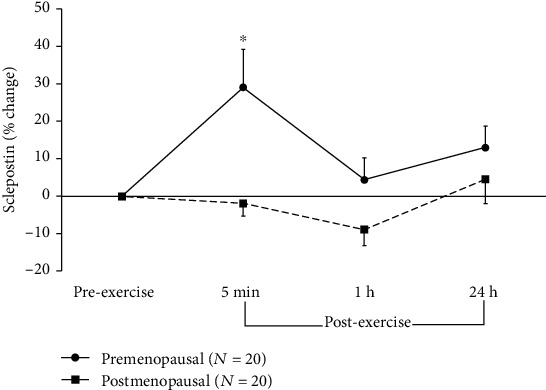
Percent changes (mean ± SEM) in serum concentrations of sclerostin from rest (preexercise) to after plyometric exercise in young, premenopausal women and in older, postmenopausal women. ∗ denotes significant differences from preexercise to 5 min postexercise in premenopausal women (*p* = 0.004, 95%CI = [7.6; 50.4]).

The results of the two-way mixed ANOVA for sclerostin showed a significant main effect for time (*F* = 4.82; *p* = 0.009, *η*_p_^2^ = 0.11), reflecting an overall increase in sclerostin over time, and a group effect (*F* = 29.34; *p* = 0.000, *η*_p_^2^ = 0.44), reflecting 31 to 41% higher concentrations in the postmenopausal women compared to their premenopausal counterparts across time (Figure 1). In addition, there was a significant group-by-time interaction, which reflects a significant 29% increase from pre- to 5 min postexercise in the premenopausal group that is not apparent in the postmenopausal group (Figure 1).

**Figure 2:**
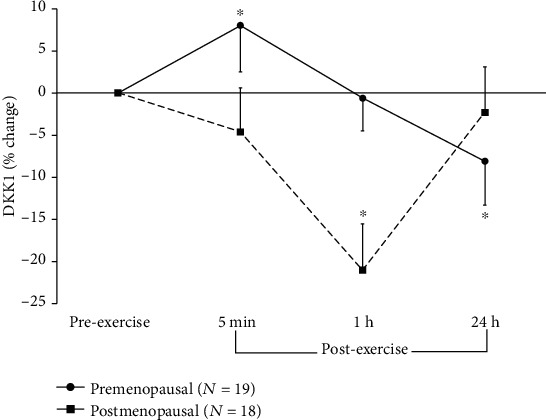
Percent changes (mean ± SEM) in serum concentrations of dickkopf-1 (DKK1) from rest (preexercise) to after plyometric exercise in young, premenopausal women and in older, postmenopausal women. ∗ denotes significant differences from preexercise to 5 min (*p* = 0.001, 95%CI = [−3.6; 19.7]) and 24 h (*p* = 0.036, 95%CI = [−19.1; 2.8]) postexercise in premenopausal women and from preexercise to 1 h postexercise (*p* = 0.000, 95%CI = [−32.6; −9.5]) in postmenopausal women.

Significant main effects for time (*F* = 7.17; *p* = 0.000, *η*_p_^2^ = 0.17) and group (*F* = 6.83; *p* = 0.013, *η*_p_^2^ = 0.16) and a significant group-by-time interaction (*F* = 7.15; *p* = 0.000, *η*_p_^2^ = 0.17) were found for DKK1. Throughout the 24 h, DKK1 was significantly higher in the premenopausal women across time (from 19% preexercise to 34% 1 h postexercise). Overall, DKK1 decreased following plyometric exercise. However, based on the group-by-time interaction, the timing of the response was different between groups. In premenopausal women, DKK1 increased immediately postexercise, then progressively decreased to 8% below preexercise levels 24 h postexercise (Figure 2). In postmenopausal women, DKK1 begun to decrease immediately postexercise, at 1 h postexercise was 21% below its preexercise levels, and at 24 h was near baseline levels (Figure 2).

**Figure 3:**
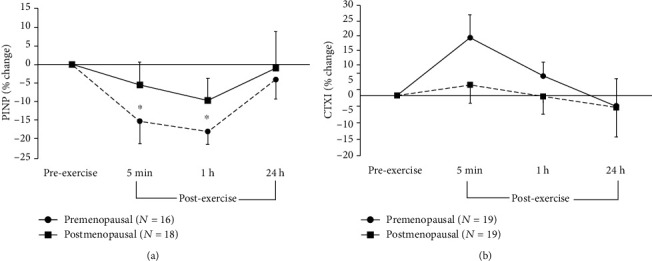
Percent changes (mean ± SEM) in serum concentrations of (a) procollagen type I amino-terminal propeptide (PINP) and (b) C-terminal crosslinking telopeptides of type I collagen (CTXI) from rest (preexercise) to after plyometric exercise in young, premenopausal women and in older, postmenopausal women. ∗ denotes significant differences in PINP from preexercise to 5 min (*p* = 0.035; 95%CI = [−18.7; −1.2]) and 1 h (*p* = 0.035; 95%CI = [−20.7; −6.2]) postexercise in both groups combined.

**Figure 4:**
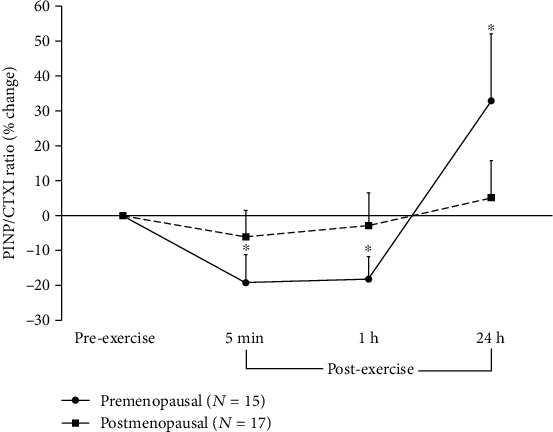
Percent changes (mean ± SEM) in the ratio of procollagen type I amino-terminal propeptide to C-terminal crosslinking telopeptides of type I collagen (PINP/CTXI) at rest and in response to plyometric exercise in young, premenopausal women and in older, postmenopausal women. ∗ denotes significant differences from preexercise to 5 min (*p* = 0.034; 95%CI = [−36.6; −1.7]), 1 h (*p* = 0.014; 95%CI = [−32.0; −4.3]), and 24 h (*p* = 0.032; 95%CI = [−8.7; +74.5]) postexercise in premenopausal women.

PINP showed a significant effect for time (*F* = 5.69; *p* = 0.003, *η*_p_^2^ = 0.15) with no significant effect for group and no group-by-time interaction. The time effect reflected a small but significant decrease from pre- to 5 min and 1 h postexercise (-15% and -18%, respectively) in both groups combined (Figure 3). CTXI showed a significant main effect for group (*F* = 10.4; *p* = 0.003, *η*_p_^2^ = 0.22), reflecting that postmenopausal women had 38 to 49% significantly higher CTXI levels than premenopausal women across time, but no significant effect for time and no group-by-time interaction (Figure 3). For the PINP/CTXI ratio, there was a significant main effect for time (*F* = 6.29; *p* = 0.002, *η*_p_^2^ = 0.18) and for group (*F* = 5.72; *p* = 0.023, *η*_p_^2^ = 0.22) reflecting a 32% lower ratio in the postmenopausal women. There was also a significant group-by-time interaction (*F* = 10.4; *p* = 0.049, *η*_p_^2^ = 0.08), which reflects that in premenopausal women, the PINP/CTXI ratio significantly decreased from preexercise to 5 min (-19%) and 1 h (-18%) postexercise; then, it was significantly 32% higher than preexercise at 24 h, with no significant changes in the postmenopausal group (Figure 4).

At rest, i.e., preexercise, CTXI was positively correlated with sclerostin (*r* = 0.33, *p* = 0.04) and negatively correlated with estradiol (*r* = −0.37, *p* = 0.04) while the PINP/CTXI ratio was positively correlated with estradiol (*r* = 0.58, *p* = 0.002). In addition, there were significant negative correlations between sclerostin and energy intake relative to body mass (*r* = −0.35, *p* = 0.024), protein intake relative to body mass (*r* = −0.34, *p* = 0.03), and calcium intake relative to body mass (*r* = −0.31, *p* = 0.045). There were no significant correlations for resting PINP and DKK1 or between the absolute changes from pre- to 5 min postexercise for sclerostin, DKK1, PINP, CTXI, and PINP/CTXI ratio.

## 4. Discussion

To our knowledge, this is the first study to compare the circulating levels of Wnt signaling-related osteokines and bone markers at rest and in response to plyometric exercise between pre- and postmenopausal women. The postmenopausal women had 41% higher resting sclerostin and 44% higher resting CTXI than the premenopausal women, which despite their 19% lower DKK1, overall suggests an upregulation of bone resorption postmenopause. This upregulation, which leads to an overall negative formation-to-resorption balance, may be attributed to the lower estrogen, characteristic in postmenopausal women, which is supported by the observed positive correlation between the PINP/CTXI ratio and estrogen in our study. Higher resting sclerostin was also correlated with higher CTXI and lower energy, protein, and calcium intake, which is in line with the association between sclerostin and bone resorption. The bone marker and osteokine responses to the plyometric exercise followed a similar pattern in the two groups, with some differences in the time course of these responses. Sclerostin increased from pre- to 5 min postexercise in premenopausal women, which was not evident in the postmenopausal group. This initial increase in sclerostin may reflect an overall stimulation of bone turnover, with an initial decrease in the formation-to-resorption ratio, but a subsequent significant increase in the ratio 24 h postexercise in pre- but not the postmenopausal women. These exercise-induced changes in bone biomarkers can potentially unveil the mechanisms by which exercise affects bone homeostasis and the role that menopause plays in altering the response of bone to different intensities and modes of exercise in aging women. The understanding of such mechanisms can lead to clinical recommendations which are population specific.

The resting concentrations of both PINP and CTXI fall within the reference intervals for their age group [33]. However, while CTXI was significantly higher in the postmenopausal group, PINP was not significantly different between groups. The higher PINP/CTXI ratio in the premenopausal group, although it did not reach significance, is consistent with a recent study reporting no differences in CTXI but significantly lower PINP/CTXI ratio in postmenopausal women with low bone mineral density compared to those with normal bone mineral density [9]. In addition, the significant correlation between the PINP/CTXI ratio and estrogen (*r* = 0.58, *p* = 0.002) supports previous studies suggesting that the decline in circulating estrogen postmenopause leads to a negative change in the balance of bone remodeling [3, 4].

The higher levels of sclerostin in postmenopausal women could also be associated with their lower circulating levels of estradiol as previously suggested [23, 34–36]. Indeed, lower circulating estradiol and higher levels of sclerostin have been previously reported, which is indicative of an inverse association between estradiol and sclerostin [2, 23, 24, 34, 35, 36]. In contrast to sclerostin, DKK1 was lower in the postmenopausal group compared to their younger counterparts, which is inconsistent with previous findings in older women compared to younger women [37] and in premenarcheal girls, who also have lower estradiol levels, compared to postmenarcheal girls [30]. Based on these previous studies, one would expect the postmenopausal women to have higher levels of serum DKK1. However, there are no studies directly comparing circulating DKK1 between younger and older adults. Nevertheless, the higher resting sclerostin accompanied by higher CTXI in the postmenopausal women is in line with an overall catabolic formation-to-resorption cycle in this group. In addition, sclerostin's negative association with energy, protein, and calcium intake may be clinically important, as it implicates nutrition in the downregulation of Wnt signaling, which can negatively affect the bone remodeling cycle over time.

Based on previous findings [26–29] in other populations, we predicted that sclerostin would increase postexercise in both groups of women. However, no exercise-induced changes in sclerostin were observed in our postmenopausal group. Premenopausal women, on the other hand, did show an exercise-induced increase 5 min postexercise in sclerostin, which dropped to baseline levels 1 h later. This is consistent with previous studies in young women that have shown circulating levels of sclerostin to transiently increase following a single bout of both running and cycling [26, 27]. Furthermore, similarly to aged young men, sclerostin was also shown to increase from pre- to 5 min postplyometric exercise [28], as well as following running and cycling [29]. This consistency across studies of a similar response in circulating sclerostin in young men and women is independent of modality of exercise. Currently, why and how circulating sclerostin is released during exercise is unclear, but it has been previously suggested that the postexercise increase in serum sclerostin may be due to release of previously synthesized sclerostin from osteocytes, rather than an increase in sclerostin's gene expression [26]. In addition, exercise-induced increased blood flow to the bone could facilitate the release of such previously synthesized sclerostin, presenting as an immediate, transient increase in circulating sclerostin levels postexercise [38]. However, the absence of a similar increase in the postmenopausal group questions this possibility. It is possible that the acute increase seen 5 min postexercise in sclerostin does not necessarily hold a catabolic paracrine significance for the bone, especially when it is combined with a later increase in the PINP/CTXI ratio in the same group of younger women. There are other influencing factors that may be contributing to the transient postexercise increase of sclerostin that cannot be ignored. For example, muscle stress and inflammation caused by exercise may contribute to the postexercise increase in sclerostin [39]. It is also possible that the transient postexercise increase in circulating sclerostin is purposeful and has to do with its potential endocrine-like effect on other tissues, specifically adipose tissue, where it has been shown to contribute, at least partially, to an increase in beige adipogenesis *in vitro* and *in vivo*, also suggesting that it may have metabolic effects on adipocytes and other metabolic tissues [40]. These proposed causes and influences of sclerostin's response to exercise cannot be confirmed by the current study. Furthermore, the nonresponse seen in the postmenopausal women may be related to their higher resting values, suggesting a ceiling effect that requires further research into the causal influences and mechanisms, especially in aging populations. An alternative explanation as to why there might not be the same increase in sclerostin among postmenopausal women may be aging-induced changes in the shape and density of the osteocyte lacuna-canalicular network. Specifically, there is evidence that the osteocyte lacunae become smaller and more spherical and their number density is reduced with aging. These changes in the morphology and density of osteocyte lacuna-canalicular network could lead to alterations in the osteocyte mechanosensitivity and response to mechanical loading [41]. It is possible that postmenopausal bones synthesize sclerostin but have fewer cells or degraded infrastructure for releasing this sclerostin after exercise.

Compared to preexercise levels, serum DKK1 was significantly lower 24 h postexercise in premenopausal women and 1 h postexercise in postmenopausal women, indicating a later response to exercise in the younger group. An overall exercise-induced decrease in DKK1 seems to be consistent with findings in other female age groups, as a postexercise decrease was also observed in pre- and postmenarcheal girls [30]. However, as no other study to date has assessed the DKK1 response to exercise, more research is needed to explain the origin of the observed exercise-induced decrease in DKK1.

CTXI did not significantly change immediately postexercise in either group of women. Previously, no change postexercise was seen in serum CTXI in a group of similarly aged women in both a running trial and a cycling trial [27]. However, in young men, we found CTXI to increase immediately after high-intensity interval running and cycling [29] while others have found CTXI to decrease 2 h following both plyometric training [16] and treadmill exercise [17]. It seems, therefore, that the CTXI response to exercise is dependent on sex, but since we did not find a group effect, it does not seem to be affected by menopause or age. We did not draw blood between 1 h and 24 h postexercises in the present study. Therefore, it is possible that we may have missed a reduction in CTXI in the hours following the exercise bout. We also found PINP to significantly decrease at 5 min and 1 h postexercise in both groups of women, which is consistent with a previously reported postexercise decrease in both male and female athletes after a cycling trial [13]. Interestingly, the PINP/CTXI ratio only changed in the premenopausal women, decreasing 5 min and 1 h postexercise due to the early reduction in PINP, then significantly increasing 24 h postexercise due to the progressive decline in CTXI. These changes in the ratio suggest that whatever the dynamic changes in the individual bone markers following the exercise are, there was an overall bone response in the premenopausal women that was not evident in the postmenopausal women. Thus, more investigation is needed to determine the nature and time course of the bone turnover responses to various exercise modalities across the lifespan.

This study had several strengths. All visits were scheduled in the morning, allowing us to control for circadian rhythm variability in bone markers. Additionally, we took measures to account for nutritional factors, including having fasting blood samples on both days of the experiment and providing participants only with a standardized breakfast between the preexercise and the first two (5 min and 1 h) postexercise blood samples. Another strength is the body composition in the two groups. Thus, there were no confounding issues of excess adiposity.

The main limitation of this study is the absence of a nonexercise control trial performed by all participants as part of the original experimental design. However, a consequent addition of a nonexercise control trial in a subset of 5 participants confirmed that resting levels of all the measured markers can be considered stable during the morning hours. In fact, we have performed a series of control trials in different age groups of males and females and confirmed that the increases found in sclerostin postexercise were 4-5 times higher than their intraindividual variability in the absence of exercise [27, 29]. Another limitation is that a habitual diet was not controlled between the 1 h and 24 h blood draws, so it is possible that some participants consumed a higher amount of energy, proteins, and/or calcium. Such differences could have affected the results of PINP/CTXI, which are known to be influenced by food consumption. However, we have no reason to believe that such an effect would have been different between the two groups. Additionally, PINP and DKK-1 are not bone-specific, so it is possible that circulating levels may be indicative of other functions and/or other tissues. Finally, although our sample size was similar or larger to that of previous studies investigating bone marker response to exercise [14–17, 26–30] and significance was reached in many of the variables, we used G∗Power (version 3.1) to calculate power posteriori. We found that for a large effect size (partial *η*^2^ = 0.15 for ANOVA) and a significance level set at 0.05, the calculated power for 40 participants was 59%. Thus, it is possible that our modest sample size (*N* = 40) may have played a role in limiting the significance of the statistical comparisons conducted.

In summary, this study found differences in bone markers and Wnt-related osteokines between younger premenopausal and older postmenopausal women both at rest and in response to an acute bout of plyometric exercise. In premenopausal women, sclerostin significantly increased transiently 5 min postexercise while DKK1 decreased 24 h after exercise. In postmenopausal women, there were no exercise-induced changes in sclerostin, but DKK1 had an earlier drop 1 h after exercise. In addition, despite the similar responses in the individual markers, the PINP/CTXI ratio significantly increased 24 h following the exercise in the premenopausal group, reflecting an overall osteogenic effect of the plyometric protocol, which was not evident in the postmenopausal women. These findings are meaningful and advance our knowledge about markers of bone turnover and osteokines in response to exercise, suggesting that age, and specifically menopause, is an important factor in bone's response to plyometric exercise in women. Based on these results, plyometric exercise is an effective way to elicit bone-specific anabolic effects in younger women and should be included and encouraged in exercise programs designed for this age group. However, further research is needed to understand the mechanisms involved and whether the changes in markers are physiologically relevant to changes in bone structure, density, or remodeling. In addition, an exercise training intervention is needed to examine whether the responses found in this acute exercise study bear implications to longer-term bone turnover, particularly in older women. Based on the results of the present study, the effectiveness of such a program in postmenopausal women is less clear. Thus, given the safety implications of high impact exercises, the inclusion of such an exercise program for postmenopausal populations is unjustified at this time.

## Figures and Tables

**Table 1:** Physical and nutritional characteristics of pre- and postmenopausal women (mean ± SEM).

Variable	Premenopausal (*N* = 20)	Postmenopausal (*N* = 20)	Δ (*p* value)
Age (years)	23.1 ± 0.5	57.9 ± 1.0	*p* < 0.001^∗^
Height (cm)	166 ± 1.9	164 ± 1.6	*p* = 0.468
Body mass (kg)	62.6 ± 1.8	65.6 ± 2.0	*p* = 0.280
Body fat (%)	24.7 ± 1.2	31 ± 1.9	*p* = 0.008^∗^
Leisure Time Physical Activity (score)	75.5 ± 9.1	70.6 ± 9.0	*p* = 0.712
Energy intake (kJ·kg^−1^·day^−1^)	113 ± 8.0	97.5 ± 7.9	*p* = 0.353
Protein intake (g·kg^−1^·day^−1^)	1.1 ± 0.09	0.9 ± 0.07	*p* = 0.311
Calcium intake (mg·day^−1^)	1014 ± 103	883 ± 91	*p* = 0.349

∗ denotes significant difference.

**Table 2:** Serum concentrations (mean ± SEM) and percent coefficient of variation (%CV) of Wnt-related osteokines and bone turnover markers across time during the control, nonexercise session in a subgroup of 3 premenopausal and 5 postmenopausal women.

Marker	Baseline	30 min	60 min	%CV
Sclerostin (pg/mL)	123.9 ± 42.6	135.1 ± 50.1	134.4 ± 53.7	9.4%
DKK1 (pg/mL)	1.43 ± 0.34	1.39 ± 0.38	1.41 ± 0.35	5.3%
PINP (ng/mL)	28.1 ± 1.9	31.1 ± 1.9	28.5 ± 2.3	10.1%
CTXI (ng/mL)	0.26 ± 0.04	0.29 ± 0.04	0.31 ± 0.05	9.5%

DKK1: dickkopf-1; CTXI: C-terminal crosslinking telopeptides of type I collagen; PINP: procollagen type I amino-terminal propeptide.

**Table 3:** Resting (preexercise) concentrations of Wnt-related osteokines, bone turnover markers, and estradiol in pre- and postmenopausal women. Data are the mean ± SEM (*N*).

Marker	Premenopausal women	Postmenopausal women	*p* value
Sclerostin (pg/mL)	240.9 ± 35.8 (*N* = 20)	410.5 ± 27.7 (*N* = 20)	<0.001^∗^
DKK1 (pg/mL)	2395 ± 137 (*N* = 19)	1949 ± 178 (*N* = 18)	0.049^∗^
PINP (ng/mL)	32.9 ± 2.1 (*N* = 16)	35.7 ± 2.8 (*N* = 18)	0.422
CTXI (ng/mL)	0.28 ± 0.03 (*N* = 19)	0.52 ± 0.29 (*N* = 19)	0.001^∗^
PINP/CTXI (ratio)	114.09 ± 8.82 (*N* = 15)	86.65 ± 15.47 (*N* = 15)	0.09
Estradiol (pg/mL)	97.2 ± 12.4 (*N* = 16)	67.4 ± 4.7 (*N* = 17)	0.035^∗^

∗ denotes significant difference. DKK1: dickkopf-1; CTXI: C-terminal crosslinking telopeptides of type I collagen; PINP: procollagen type I amino-terminal propeptide. Note: raw data are shown here for all markers; log transformed data were used for the independent *t*-tests for PINP, CTXI, and PINP/CTXI.

## Data Availability

The blood and statistical data used to support the findings of this study are restricted by the Brock University Research Ethics Board to protect participant privacy. The data are available upon request from the corresponding author for researchers who meet the criteria for access to confidential data.
